# n-Butylidenephthalide (BP) Maintains Stem Cell Pluripotency by Activating Jak2/Stat3 Pathway and Increases the Efficiency of iPS Cells Generation

**DOI:** 10.1371/journal.pone.0044024

**Published:** 2012-09-07

**Authors:** Shih-Ping Liu, Horng-Jyh Harn, Ying-Jiun Chien, Cheng-Hsuan Chang, Chien-Yu Hsu, Ru-Huei Fu, Yu-Chuen Huang, Shih-Yin Chen, Woei-Cherng Shyu, Shinn-Zong Lin

**Affiliations:** 1 Graduate Institute of Basic Medical Science, China Medical University, Taichung, Taiwan; 2 Center for Neuropsychiatry, China Medical University Hospital, Taichung, Taiwan; 3 Department of Pathology, China Medical University Hospital, Taichung, Taiwan; 4 Graduate Institute of Immunology, China Medical University, Taichung, Taiwan; 5 Genetics Center, Department of Medical Research, China Medical University Hospital, Taichung, Taiwan; 6 Graduate Institute of Chinese Medical Science, China Medical University, Taichung, Taiwan; University of Medicine and Dentistry of New Jersey, United States of America

## Abstract

In 2006, induced pluripotent stem (iPS) cells were generated from somatic cells by introducing Oct4, Sox2, c-Myc and Klf4. The original process was inefficient; maintaining the pluripotency of embryonic stem (ES) and iPS cell cultures required an expensive reagent–leukemia induced factor (LIF). Our goal is to find a pure compound that not only maintains ES and iPS cell pluripotency, but also increases iPS cell generation efficiency. From 15 candidate compounds we determined that 10 µg/ml n-Butylidenephthalide (BP), an Angelica sinensis extract, triggers the up-regulation of Oct4 and Sox2 gene expression levels in MEF cells. We used ES and iPS cells treated with different concentrations of BP to test its usefulness for maintaining stem cell pluripotency. Results indicate higher expression levels of several stem cell markers in BP-treated ES and iPS cells compared to controls that did not contain LIF, including alkaline phosphatase, SSEA1, and Nanog. Embryoid body formation and differentiation results confirm that BP containing medium culture was capable of maintaining ES cell pluripotency after six time passage. Microarray analysis data identified PPAR, ECM, and Jak-Stat signaling as the top three deregulated pathways. We subsequently determined that phosphorylated Jak2 and phosphorylated Stat3 protein levels increased following BP treatment and suppressed with the Jak2 inhibitor, AG490. The gene expression levels of cytokines associated with the Jak2-Stat3 pathway were also up-regulated. Last, we used pou5f1-GFP MEF cells to test iPS generation efficiency following BP treatment. Our data demonstrate the ability of BP to maintain stem cell pluripotency via the Jak2-Stat3 pathway by inducing cytokine expression levels, at the same time improving iPS generation efficiency.

## Introduction

Stem cells are currently being used for many clinical therapeutic purposes. For example, the combination of hematopoietic stem cells (HSCs) and transplanted bone marrow is applied to treat leukemia, hemophilia, and anemia. Cells that respond to ischemia or injuries and are part of revascularization processes are known as mesenchymal stem cells (MSCs) [1]. Embryonic stem cells (ESCs), which are pluripotent cells derived from the inner cell masses of mammalian blastocysts, are capable of differentiating into the endodermal, mesodermal, and ectodermal cells of embryos [2]. ESCs are viewed as having significant potential for clinical cell therapies due to their ability to self-renew and differentiate into a wide range of specialized cell types [3]. However, they have two major drawbacks for therapeutic use: immune rejection and challenges based on ethical concerns.

Induced pluripotent stem (iPS) cells can be generated from human and mice fibroblasts to which four genes have been introduced: Oct4, Sox2, c-Myc and Klf4 [4], [5]. They are similar to ESCs in terms of proliferation, morphology, gene expression, surface antigens, the epigenetic status of pluripotent cell-specific genes, and telomerase activity. ES and iPS cell pluripotency gives them exciting potential for use in tissue repair and replacement therapies [6], but the inefficiency of reprogramming primary human cells makes it difficult to generate patient-specific iPS cells from a small starting population [7]. In addition, maintaining ES and/or iPS cell pluripotency requires treatment with leukemia inhibitory factor (LIF), an expensive reagent. LIF signaling (via Jaks) involves the activation of Stat3 (a signal transducer and activator of transcription 3) [8], which is essential for LIF-dependent ES cell self-renewal [9]. LIF transmits signals via LIF receptors and gp130, a co-receptor of the IL-6 cytokine family that also includes IL-11, CNTF, and OSM. [10], [11], [12], [13], [14] The gp130 and LIF receptors lack kinase catalytic domains, but they are capable of binding to and activating one or more members of the Jak-Stat tyrosine kinase family [15], [16], [17]. The cytokine superfamily uses the Jak-Stat pathway as a major signaling pathway into cell nuclei [18].

Angelica sinensis (called *danggui* in Chinese), one of the most commonly used traditional Chinese medicines, is variously prescribed as a tonic, hemopoetic, spasmolytic, and analgesic [19]. n-Butylidenephthalide (BP), a compound derived from Angelica sinensis chloroform extract, has been identified as having a strong antitumoral effect, arresting the growth and apoptosis of malignant brain tumors in vitro and in vivo [20], [21]. However, while these findings indicate that BP holds potential as an anti-cancer compound for clinical applications, little is known about BP function in terms of stem cell activity.

Our goal in this study was to determine whether a pure compound extracted from a traditional Chinese medicine is capable of maintaining ES and iPS cell pluripotency while increasing iPS cell generation efficiency. Our main finding is that BP is capable of maintaining ES and iPS cell pluripotency via Jak2 and Stat3 activation. We also determined that BP treatment increased iPS cell generation efficiency.

## Results

### MTT Assays for BP Treatment

**Figure 1:**
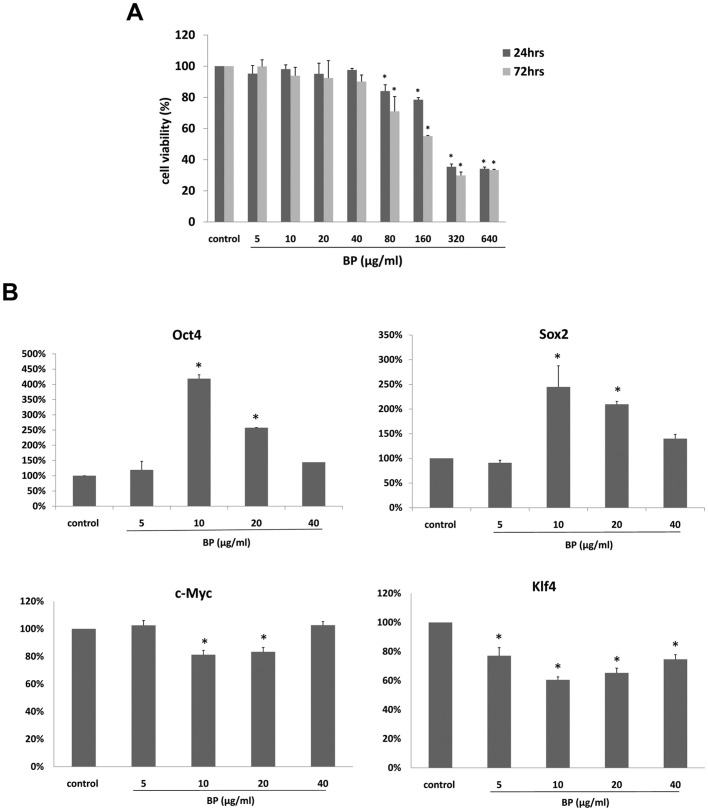
Cell viability and iPS cell related gene expression levels in MEF cells treated with BP. (A) MTT assay of MEF cells treated with various concentrations of BP. (B) Oct4, Sox2, c-Myc and Klf4 gene expression levels in MEF cells treated with various concentrations of BP (real-time PCR).

MTT assays were used to identify MEF cell viability following BP treatment. According to data from tests using eight concentrations of BP (5, 10, 20, 40, 80, 160, 320 and 640 µg/ml), cell survival rates significantly decreased in the 80, 160, 320 and 640 µg/ml treatment groups 24 h and 72 h post-treatment (Fig. 1A). We therefore selected the 5, 10, 20 and 40 µg/ml BP concentrations for our experiments.

### BP Treatment Resulted in Oct4 and Sox2 Gene Expression Up-Regulation

Our data indicated significant up-regulation of Oct4 and Sox2 gene expression levels in MEF cells following treatment with BP, especially in the 10 µg/ml treatment group (Fig. 1B). The effect decreased as concentration increased to 20 and 40 µg/ml. Oct4 and Sox 2 expression levels were higher than in the solvent control group; in contrast, c-Myc and Klf4 gene expression levels were not.

### BP Treatment Maintained ES and iPS Cell Self-Renewal

**Figure 2:**
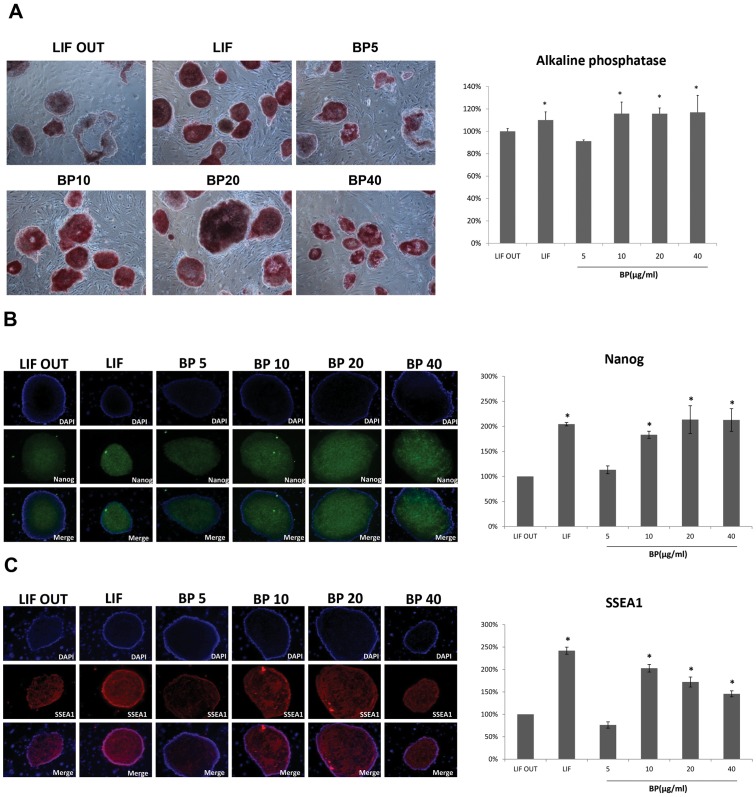
Stem cell markers staining in ES cells treated with BP. (A) Alkaline phosphatase staining ES cells treated with various concentrations of BP and the quantities of AP-positive clones in ES cells. (B) SSEA1 immunocytochemistry in ES cells treated with various concentrations of BP and the quantities of SSEA1 expression levels in ES cells. (C) Nanog immunocytochemistry in ES cells treated with various concentrations of BP and the quantities of SSEA1 expression levels in ES cells. *p < 0.05 versus control.

After detecting Oct4 and Sox2 gene expression up-regulation following BP treatment, we performed tests to determine whether BP treatment also maintained ES and iPS cell self-renewal and pluripotency as an alternative to LIF. Alkaline phosphatase (AP), Nanog, and SSEA1 were used to identify ES and iPS cell pluripotency. According to results from our ES cell analyses, quantities of AP-positive clones in ES cells treated with 10, 20 or 40 µg/ml of BP were all significantly higher than in the controls that did not contain LIF (LIF OUT control) (Fig. 2A). However, quantities of AP-positive clones in ES cells treated with 5 µg/ml BP were lower compared to the LIF OUT cells (Fig. 2A). In addition, data for Nanog and SSEA-1 levels in ES cells indicated that those treated with 10, 20 or 40 µg/ml BP had higher quantities compared to the LIF OUT control cells (Figs 2B and C).

**Figure 3:**
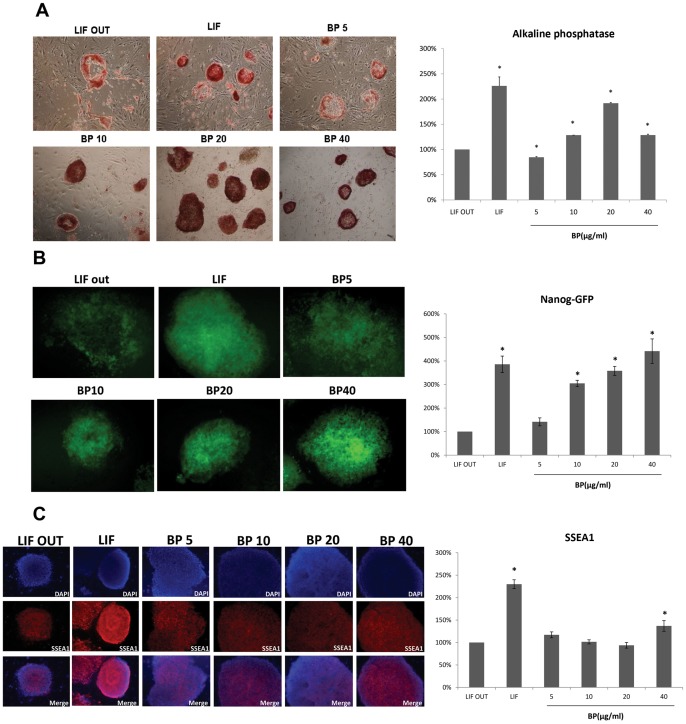
Stem cell markers staining in iPS cells treated with BP. (A) Alkaline phosphatase staining iPS cells treated with various concentrations of BP and the quantities of AP-positive clones in iPS cells. (B) SSEA1 immunocytochemistry in iPS cells treated with various concentrations of BP and the quantities of SSEA1 expression levels in iPS cells. (C) Nanog immunocytochemistry in iPS cells treated with various concentrations of BP and the quantities of SSEA1 expression levels in iPS cells. *p < 0.05 versus control.

Mouse iPS cells (from Nanog-GFP transgenic mice) were obtained from the laboratory of Dr. Shinya Yamanaka. [5] AP staining analysis data were similar to ES cell results, with quantities of AP-positive clones in 10, 20 and 40 µg/ml BP-treated iPS cells being significantly greater compared to the LIF OUT control cells (Fig. 3A). As shown in Fig. 3B, GFP signaling (Nanog expression) in the 10, 20 and 40 µg/ml BP-treated cells was higher than in the LIF OUT controls. The SSEA-1 stem cell marker staining showed that 40 µg/ml BP-treated cells was higher than in the LIF OUT controls (Fig. 3C). Combined, the data indicate the ability of BP to maintain ES and iPS cell self-renewal and pluripotency.

### Embryoid Body Formation and Differentiation

**Figure 4:**
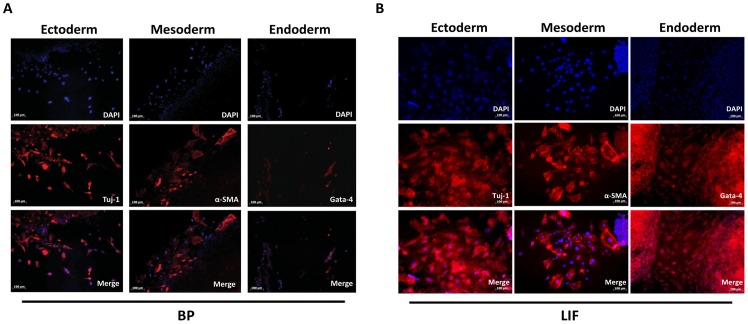
Embryoid body-mediated differentiation of ES cells passaged three times in (A) BP containing medium (replaced LIF) and (B) LIF containing medium as control. Immunofluorescent staining of Tuj1 (ectoderm marker), α-smooth muscle actin (α-SMA, mesoderm marker) and Gata4 (endoderm marker). Nuclei were stained with DAPI (blue).

In another test designed to determine the ability of BP to serve as a LIF substitute in ES cell cultures, embryoid body formation was used to determine ES cell pluripotency by passaging cells six times in culture containing BP (LIF replacement) medium. We observed differentiation initiated by ES cells after adding the embryoid bodies to cell culture plates. Immunofluorescent staining was used to detect cells that were positive for Tuj1 (ectoderm marker), α-smooth muscle actin (α-SMA, mesoderm marker), and Gata4 (endoderm marker). As shown in Fig. 4A, we observed ES cells differentiation in all three germ layer cell types, including neuronal cells (Tuj1), hepatocyte (Gata4) and α-smooth muscle actin (muscle cells). The LIF control differentiation in all three germ layer cell types was showed in Fig. 4B. These data also suggest that BP can serve as a substitute for LIF in ES cell cultures to maintain cell pluripotency after passaging.

### Microarray Analysis of Genes Involved in Various Pathways in BP-Treated MEF Cells

Microarray analyses were performed to evaluate gene expression profiles in various pathways in MEF cells treated with 10 µg/ml BP, 40 µg/ml BP, and DMSO (control). All data were submitted to GEO system and the accession numbers are MEF (GSM886449), MEF cells treated with 10 µg/ml BP (GSM886450), and MEF cells treated with 40 µg/ml BP (GSM886451), respectively. A Babelomics web tool and KEGG pathways database were used as part of this procedure. Numbers and percentages of significantly deregulated genes with known biological functions are shown in Table 1. Higher percentages of deregulated genes post-BP treatment were observed in PPAR, ECM-receptor interaction, and Jak-Stat signaling pathways. Since the Jak-Stat signaling pathway is the most important in terms of stem cell maintenance and renewal, we selected it to study stem cell self-renewal and pluripotency maintenance mechanisms.

**Table 1:** Numbers of significantly deregulated genes with known biological functions classified according to KEGG and Babelomics databases.

	BP10				BP40			
Function/Pathway	I	D	No.	%	I	D	No.	%
**Signal transduction**								
PPAR signaling pathway	14	14	28/74	37.8	12	14	27/74	36.5
ECM-receptor interaction	3	18	21/74	28.4	3	16	19/74	25.7
JAK-STAT signaling pathway	9	30	39/140	27.9	8	27	35/140	25
Calcium signaling pathway	4	28	32/146	21.9	2	30	32/146	21.9
TGF-beta signaling pathway	3	2	5/45	11.1	5	1	6/45	13.3
MAPK signaling	7	17	24/227	10.6	5	19	24/227	10.6
Wnt signaling pathway	2	5	7/66	10.6	0	6	6/66	9.1
Insulin signaling pathway	3	7	10/102	9.8	2	3	5/102	4.9
VEGF signaling pathway	2	0	2/31	6.5	0	0	0/31	0
**Cell proliferation**								
Cell communication	3	17	20/92	21.7	2	18	20/92	21.7
Cell cycle	0	4	4/163	2.5	0	6	6/163	3.7
**Metabolism**								
Lipid metabolism	3	15	18/109	16.5	2	12	14/109	12.8
Amino acid metabolism	0	1	1/23	4.3	0	2	2/23	8.7
**Cell adhesion**								
Cell adhesion molecules	13	20	33/150	22	13	21	34/150	22.7
Tight junction	2	7	9/66	13.6	2	9	11/66	16.7
Focal adhesion	3	16	19/173	11	2	20	22/173	12.7
**Apoptosis**	3	8	11/94	11.7	1	6	7/94	7.4

I: number of up-regulated genes; D: number of down-regulated genes.

### Phosphorylated Jak2 and Stat3 Levels in BP-Treated ES Cells

**Figure 5:**
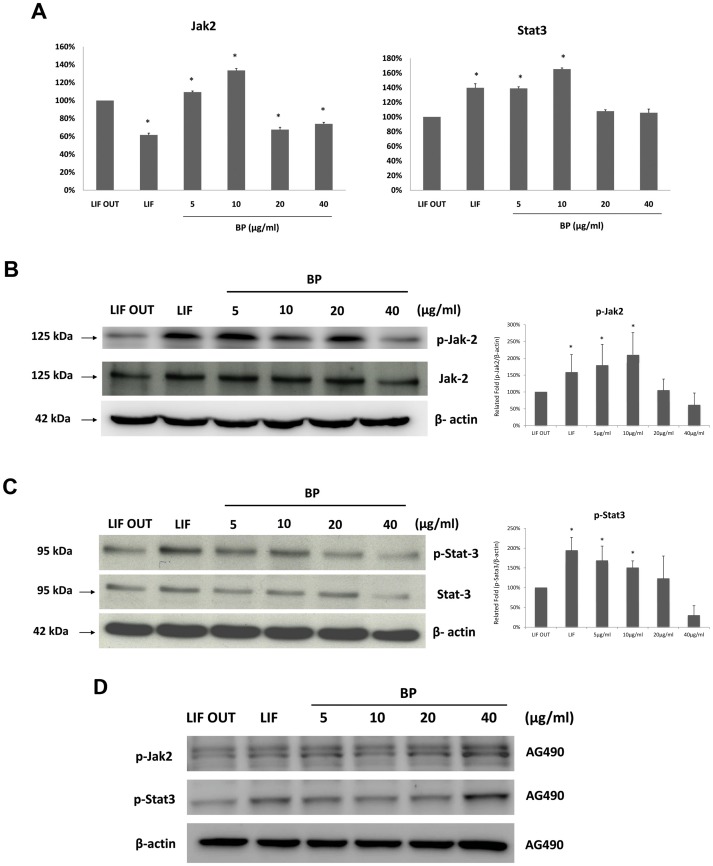
Jak2 and Stat3 expression levels in BP-treated ES Cells. (A) Jak2 and Stat3 gene expression levels in ES cells treated with various concentrations of BP (real-time PCR). (B) Jak2 and phosphorylated-Jak2 protein levels in ES cells treated with BP (western blot). (C) Stat3 and phosphorylated-Stat3 protein levels in ES cells treated with BP (western blot). (D) phosphorylated-Jak2 and phosphorylated-Stat3 protein levels in ES cells treated with BP and Jak2 inhibitor, AG490 (western blot).

Jak2 and Stat3 are known as key Jak-Stat pathway proteins for maintaining stem cell self-renewal. We used real-time PCR and western blot assays to determine Jak2 and Stat3 gene mRNA and protein levels in BP-treated ES cells. As shown in Fig. 5A, both Jak2 and Stat3 mRNA levels significantly increased after treatment with 5 or 10 µg/ml BP. Compared to the LIF OUT controls, Jak2 and Stat3 protein levels increased in ES cells treated with 5 and 10 µg/ml BP (Figs. 5B and C). In addition, the phosphorylated Jak2 and Stat3 forms (active forms) also increased following treatment with 5 and 10 µg/ml BP. According to these data, BP maintains ES cell self-renewal by activating the Jak2-Stat3 signaling pathway. It was also observed that the Jak2 inhibitor, AG490 suppressed the phosphorylated Jak2 and phosphorylated Jak2 expression in ES cells treated with BP (Fig. 4D).

### Cytokine Regulation in BP-Treated ES Cells

**Figure 6:**
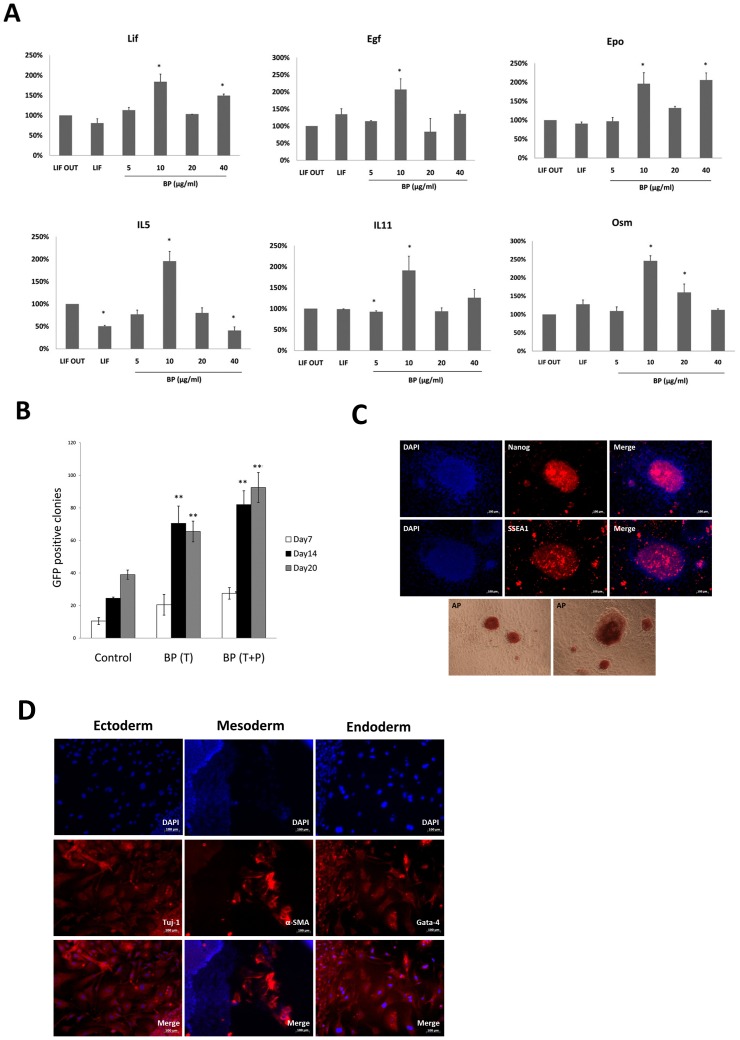
Cytokine regulation in BP-treated ES cells and characterizations of the novel iPS cells generated by adding BP (iPS-BP). (A) Real-time PCR results for cytokine gene expression levels associated with the Jak2-Stat3 pathway. Significant up-regulation of six cytokine genes was noted following BP treatment (10 µg/ml). (B) Pou5f1-GFP MEF cells were introduced with plasmids containing Oct4, Sox2, c-Myc and Klf4 to test iPS cell-generation efficiency. BP(T): treatment with BP post-transfection. BP(T+P): treated with BP post-transfection and following passage to feeder cells. Control group was introduced with plasmids containing Oct4, Sox2, c-Myc and Klf4 to generate non-BP treated iPS cells. (C) The stem cell markers (Nanog, SSEA1 and alkaline phosphotase) staining of iPS-BP cells. Nuclei were stained with DAPI (blue). (D) Immunofluorescent staining in iPS-BP cells: Gata4 (endoderm marker), α-smooth muscle actin (mesoderm marker), and Tuj1 (ectoderm marker). Nuclei were stained with DAPI (blue).

To understand why BP treatment resulted in Jak2-Stat3 signaling up-regulation, we used real-time PCR analysis to determine the mRNA levels of cytokine genes involved in the Jak2-Stat3 pathway: LIF, EGF, EPO, IL-5, IL-11 and OSM. As shown in Fig. 6A, significant up-regulation following treatment with 10 µg/ml BP was observed for LIF, EGF, EPO, IL-5, IL-11 and OSM. These data match those showing the highest levels of phosphorylated Jak2 and Stat3 following treatment with 10 µg/ml BP. Combined, the results suggest that BP is capable of increasing Jak2-Stat3-related cytokine levels, thus activating Jak2 and Stat3 proteins to maintain stem cell pluripotency.

### Improved iPS Cell Generation Efficiency

One of our primary goals was to determine if BP can be used to enhance the efficiency of iPS cell generation. We therefore used MEF obtained from Pou5f1-GFP mice to introduce plasmids containing Oct4, Sox2, c-Myc and KlF4 genes once every two days (4 times total) to generate iPS cells. As part of this process, 10 µg/ml of BP-containing medium was used to culture MEF cells 6 h post-transfection. We established two groups after seeding feeder cells on day 9: BP (T), meaning that treatment was limited to BP-only following transfection, and BP (T+P), meaning that BP treatment occurred both after transfection and after passaging feeder cells. The data show that regardless of treatment/no treatment with 10 µg/ml BP after seeding, the efficiency of iPS cell generation (GFP positive clones) significantly increased in the BP treatment group (Fig. 6B). In summary, we found evidence that BP treatment enhances iPS cell generation efficiency.

### Characterizations of the Novel iPS Cells Generated by Adding BP (iPS-BP)

iPS-BP cells generated by the method mentioned above (BP[T+P]) were determined the pluripotentcy of these novel cells. We used immunofluorescent staining to determine stem cell marker expression in iPS-BP cells. iPS-BP cells were stained with mouse antibodies against Nanog, SSEA1 and alkaline phosphotase. As shown in Fig. 6C, iPS-BP cells tested positive for all four stem cell markers. In addition, we used embryoid body formation to determine iPS-BP cell pluripotency. The results indicated that iPS-OSH cells formed embryoid bodies in low-attached plates. We observed differentiation initiated by the iPS-BP cells after placing the embryoid bodies in cell culture plates. Next, we used immunofluorescent staining to detect cells that were positive for Gata4 (endoderm marker), α-smooth muscle actin (mesoderm marker), and Tuj1 (ectoderm marker). As shown in Figure 6D, we observed iPS-BP cells differentiation in all three germ layer cells, including endoderm (Gata4), mesoderm (α-smooth muscle actin), and ectoderm (Tuj1).

## Discussion

Stem cells are capable of self-renewal and differentiation into a wide range of cell types with multiple clinical and therapeutic applications [22]. Of the stem cells we cultured, ES cells have the characteristics of pluripotency and the ability to differentiate into three primary germ layer derivatives. These are important attributes for producing healthy cells for therapeutic purposes. Induced pluripotent (iPS) cells generated from somatic cells via the introduction of four transcriptional factors (Oct4, Sox2, c-Myc and Klf4)6 positively address two important issues associated with ES cells: immune rejection and medical ethics. However, embryonic stem cells (ESCs) and iPS cells require treatment with LIF (an expensive reagent) via the Jak-Stat pathway in order to maintain pluripotency. [8] In addition, low iPS cell generation efficiency is considered an important limitation of iPS cell technology. The BP compound that we tested in this study, derived from Angelica sinensis, has potential to not only serve as a substitute for LIF, but to also improve that efficiency.

BP significantly arrests malignant brain tumor growth and apoptosis in vitro and in vivo. [20], [21] However, this anti-tumoral effect requires high concentrations of BP–between 50 and 100 µg/ml. [23], [24] Little effort has been made to determine the effects of low BP concentrations such as those used in the present study (40 µg/ml or less), or the mechanisms involved. Results from our MTT assays indicate that treatment at less than 40 µg/ml does not lead to cell death (Fig. 1A), which supports our decision to use lower concentrations in our experiments.

According to Nichols et al., pluripotent stem cell formation in mammalian embryos depends on Oct4;28 later studies revealed that members of the Sox family are also key genes in mammalian development [25]. Specifically, Oct4 and Sox2 have been identified as key proteins in maintaining stem cell pluripotency. We observed up-regulation of Oct4 and Sox2 expression levels in MEF cells following BP treatment at concentrations as low as 10 µg/ml, and were surprised to find decreases in this effect at 20 µg/ml and 40 µg/ml. We therefore used microarray analyses to determine differences in gene expression profiles at 10 µg/ml and 40 µg/ml concentrations, and found no differences between the two in terms of KEGG pathway (Table 1). Our conclusion is that BP treatment at 10 µg/ml is optimal for Oct4 and Sox2 up-regulation.

As Ezashi et al. report, human ES cells grow equally well under hypoxic and normoxic conditions [6]. Tejedo et al. reported that the exposure of LIF-deprived mESCs to low levels of NO prevent the loss of self-renewal gene expression (i.e., Oct4, Nanog and Sox2) [26]. Sasaki et al. recently described the contribution of LacdiNAc (GalNAcβ1-4GlcNAc) to mouse embryonic stem cell self-renewal via the regulation of leukemia inhibitory factor/Stat3 signaling [27]. However, none of these researchers considered the potential of natural compounds to maintain stem cell self-renewal. We used BP, an extract of Angelica sinensis, as a substitute for LIF when culturing ES and iPS cells, and then used several stem cell markers containing AP, Nanog, and SSEA-1 to determine the effects of that substitution. Third, we used embryoid body formation, a powerful method used in iPS studies for confirming stem cell pluripotency [4], [5], [28], [29], to determine ES cell pluripotency by passaging cells three times in culture containing 20 µg/ml BP medium. Our results indicate that the cells maintained pluripotency and the ability to differentiate into three germ layers.

To identify related mechanisms, we used microarray analyses to identify three major pathways affected by BP: PPAR signaling, ECM-receptor interaction, and Jak-Stat signaling. Mo et al. have described how peroxisome proliferator-activated receptors (PPARγ, a nuclear receptor transcription factor) regulates the LIF-induced growth and self-renewal of mouse ES cells via the Tyk2-Stat3 pathway [30]. We measured PPARγ levels in BP-treated and control ES cells, and failed to find any significant differences (data not shown). Focusing on the Jak-Stat signaling pathway, we observed increases in phosphorylated-Jak2 and phosphorylated-Stat3 protein levels following BP treatment (Figs. 5B and C). According to Niwa et al., [8] LIF signaling via Jak2 involves Stat3 activation, which is essential for LIF-dependent ES cell self-renewal [9]. Our data indicate that BP treatment activates Jak2 and Stat3 proteins to maintain ES cell self-renewal; furthermore, the Jak-Stat signaling pathway is activated by cytokines such as LIF, EGF, EPO, IL5, IL11 and OSM. [10], [11], [12], [13], [14] After detecting the gene expression levels of these cytokines, we observed the up-regulation of these genes following treatment with 10 µg/ml BP (Fig. 6A). This finding fits well with the observation that the highest levels of phosphorylated Jak2 and phosphorylated Stat3 resulted from BP treatment at 10 µg/ml. We determined that BP treatment increased Jak2-Stat3 related cytokine levels to activate Jak2 and Stat3 proteins, which in turn maintained stem cell pluripotency.

Finally, we introduced four factors via plasmid transfection to test our hypothesis that the BP-associated up-regulation of Oct4 and Sox2 levels also enhances iPS cell generation efficiency. Valproic acid (VPA) is one of several factors that have been tested in an effort to increase the efficiency of iPS cell generation [7]. Our results indicate that VPA treatment did not increase Oct4 and Sox2 expression, but did increase the virus insertion rate. According to Esteban et al., treatment with vitamin C increases the generation of both mouse and human iPS cells [31]. In the present study we used pou5f1-GFP MEF cells to introduce with Oct4, Sox2, c-Myc and Klf4 genes to compare iPS cell generation efficiency with and without BP treatment. Our data indicate that BP treatment produced a 5-fold to 8-fold increase, especially when treatment continued following seeding to feeder cells (Fig. 6B). Our current plans include identifying other factors combined with BP treatment that enhance safety and efficiency when generating iPS cells. In summary, our data demonstrated the ability of BP to maintain stem cell pluripotency via the Jak2-Stat3 pathway by inducing cytokine expression levels, at the same time improving iPS generation efficiency.

## Materials and Methods

### Mouse Embryonic Fibroblast Cell Cultures

Primary mouse embryonic fibroblast (MEF) cells were isolated from the 13.5 d-old embryos of C57BL/6 mice. Embryos were retrieved by Cesarean section, and internal organs, legs, and heads were removed. The remaining embryo parts were minced with fine scissors and placed in tubes containing Trypsin for cell digestion. MEF cells were cultured in DMEM (GIBCO BRL) with 10% heat-inactivated FBS (GIBCO BRL), penicillin (100 U/ml), streptomycin (100 µg/ml), non-essential amino acids (0.1 mM), and L-glutamine (2 mM) in a humidified incubator (37°C, 5% CO_2_). Experimental protocols were approved by the Institutional Animal Care and Use Committee of China Medical University (100-57-N).

### Mouse iPS and Embryonic Stem Cell Cultures

Mouse iPS cells were a gift from Dr. Shinya Yamanaka of the Riken Bioresource Center (Ibaraki, Japan) [5]. These and ES cells were cultured in DMEM (GIBCO BRL) with 15% heat-inactivated FBS (Hyclone), non-essential amino acids (0.1 mM), L-glutamine (2 mM), β-mercaptoethanol (0.2 mM) and LIF (4 ng/ml) in a humidified 37°C incubator with 5% CO_2_.

### BP Treatment

BP (Angelica sinensis extract; mol weight 188.23, 95%) was purchased from Lancaster Synthesis Ltd. (Newgate Morecambe, UK). BP was dissolved in DMSO to a concentration of 100 mg/ml and stored at −20°C until used as a stock solution. MTT assays were performed using 5, 10, 20, 40, 80, 160, 320 and 640 µg/ml concentrations. Concentrations at 5, 10, 20, and 40 µg/ml were used to determine gene expression profiles and for maintaining stem cell self-renewal.

### MTT Assays

MEF cells were re-seeded in 96-well plates and treated with different concentrations of BP. Prior to each assay, culture medium in each well was replaced by 100 µL fresh medium containing 10 µL of 5 mg/mL MTT (Sigma-Aldrich) stock solution. After 4 h of labeling cells with MTT, medium was removed and replaced with 100 µL DMSO in each well for 10 min at 37°C. Samples were mixed and absorbances read at 540 nm [32].

### Real-Time PCR and RT-PCR

TRIzol (Invitrogen, Carlsbad, CA) was used to extract total RNA from MEF and ES cells; concentrations were determined by spectrophotometry. Complementary DNA was produced from mRNA (5 µg) using a SuperScript III Reverse Transcriptase Kit (Invitrogen). Real-time PCR was performed as previously described [33], [34] to determine the gene expression levels of Oct4, Sox2, c-Myc, Klf4, Jak2, Stat3, LIF, EGF, EPO, IL5, IL-11 and OSM. Primer sequences are listed in Table 2.

**Table 2:** Primers used for real-time PCR.

Primer	Forward Sequence	Reverse Sequence	Taqman Probe
Sox2	AGGGCTGGACTGCGAACTG	TTTGCACCCCTCCCAATTC	
Myc	CATTCAAGCAGACGAGCA	CGAGTTAGGTCAGTTTATGCAC	
Klf4	CCTTTCAGTGCCAGAAGT	ACTACGTGGGATTTAAAAGTGC	
Jak2	CAATGATAAACAAGGGCAAATGAT	CTTGGCAATCTTCCGTTGCT	
Stat3	CCCCGTACCTGAAGACCAAGT	CCGTTATTTCCAAACTGCATCA	
EGF	GAGTCTGCCTGCGGATGGT	GCTGCAGGGAGGGAGACA	
EPO	CCCCCACGCCTCATCTG	TGCCTCCTTGGCCTCTAAGA	
IL5	TCCCTGCTACTCTCCCCAAA	CAACCTTCTCTCTCCCCAAGAA	
IL11	CATGCCACACCCCAAACAA	CCCCTCACCCAGGTCTACTG	
LIF	CCTACCTGCGTCTTACTCCATCA	TGTTTTCCCCAAAGGCTCAA	
OSM	CGGTCCACTACAACACCAGATG	GCGATGGTATCCCCAGAGAA	
β-actin	GTGCGTGACATCAAAGAGAAGC	TGGATGCCACAGGATTCCATAC	
Taqman-Oct4	GAGGCTACAGGGACACCTTTC	GTGCCAAAGTGGGGACCT	Roche Universal Probes 6
Taqman-β-actin	CTAAGGCCAACCGTGAAAAG	ACCAGAGGCATACAGGGACA	Roche Universal Probes 64

### Alkaline Phosphatase Staining and Indirect Immunofluorescent Antibody Assays (IFA)

Alkaline phosphatase staining was performed using a Vector Leukocyte Alkaline Phosphatase kit. For IFA, cells cultured in slides were treated with fixing solution I (4% paraformaldehyde, 400 mM sucrose in PBS) and held for 30 min at 37°C prior to treatment with fixing solution II (fixing solution I plus 0.5% Triton X-100) at room temp for 15 min. After washing with PBS, slides were treated with blocking buffer (0.5% BSA in PBS) and held at room temp for 1 hr, then washed 3 times with PBS prior to reacting with anti-Nanog (Novus) or anti-SSEA1 (Millipore) antibodies at a dilution of 1∶100 (4°C overnight or 37°C for 1 hr). Slides were then washed with cold PBS 5 times prior to reacting with FITC-conjugated anti-mouse IgG or TRITC-conjugated anti-rabbit IgG (Sigma-Aldrich) at a dilution of 1∶500. After 5 additional washings with cold PBS, slides were mounted and observed using a fluorescence microscope. DAPI (Invitrogen) was used to stain the DNA and localize the nucleus.

### Embryoid Body Formation and Differentiation

Embryoid body formation and differentiation experiments were used to determine whether BP can serve as a LIF replacement for ES cell cultures. ES cells were passaged six times onto 0.1% gelatin-coated tissue culture dishes with feeder layer and cultured with BP-containing medium. After final passages, ES cells were harvested by trypsinization and transferred to ultra-low attached culture dishes in ES medium without LIF. After 3 days, aggregated cells were placed into gelatin-coated tissue culture dishes and incubated for another 3 days before staining with anti-alpha smooth muscle actin monoclonal (Chemicon), anti-Gata4 polyclonal (Abcam), or anti-Tuj1 monoclonal antibodies (Chemicon) plus DAPI.

### DNA Microarrays

Total RNA from MEF or MEF treated with either 10 µg/ml or 40 µg/ml BP were labeled with Cy3. Samples were hybridized to Agilent Mouse G3 Whole Genome Oligo 8×60K microarrays according to the manufacturer’s instructions. Arrays were scanned with a Microarray Scanner System and data analyzed using GeneSpring GX software (both from Agilent, Santa Clara, CA).

### AG490 Treatment

AG490 was purchased from Sigma-Aldrich. AG490 was dissolved in methanol to a concentration of 5 mM and stored at −20°C until used as a stock solution. AG490 were used to inhibit the Jak2 that performed using 5 µM concentrations in the medium.

### Western Blot (WB) Assays

Western blot procedures have been described previously [34]. Rabbit anti-Jak2 (cell signaling), mouse anti-phospho-Jak2 (cell signaling), rabbit anti-Stat3 (BD), and rabbit anti-phospho-Stat3 (cell signaling) were used in this research.

### Determining iPS Cell Generation Efficiency

MEF cells isolated from Pou5f1-GFP transgenic mice (Jackson Lab) were used to determine iPS cell generation efficiency. pCX-OKS-2A and pCX-cMyc plasmid DNA (supported by Addgene) [28] were transfected into Pou5f1-GFP MEF cells once every two days (4 times total). Medium was changed to 10 µg/ml of BP-containing medium 6 h post-transfection, and held until the next transfection. On day 9, the MEF cells were passaged onto feeder cells and observed to detect ES cell-like clones. After seeding, medium was changed to iPS cell culture medium with or without 10 µg/ml BP, and the two types were separated. One type continued to be treated with 10 µg/ml BP (BP[T+P]), while the other was used with iPS cell culture medium only (BP[T]).

### Statistical Analysis

Results are expressed as mean ± SD. Student’s t-tests were used to evaluate mean differences between control and treatment groups. Data lacking normal distribution were analyzed by one-way ANOVA; statistical significance was established as p<0.05.
